# Crowned Dens Syndrome: A Challenging Diagnosis in Older Adults Presenting With Acute Neck Pain

**DOI:** 10.7759/cureus.37101

**Published:** 2023-04-04

**Authors:** Touba Naim, Dawlat Khan, Mohammad Ali, Joseph Fanciullo

**Affiliations:** 1 Internal Medicine, Avera McKennan Hospital and University Health Center, Sioux Falls, USA; 2 Internal Medicine, University of South Dakota Sanford School of Medicine, Sioux Falls, USA

**Keywords:** soft tissue calcifications, calcifications, cervical spine pain, acute neck pain, calcium pyrophosphate, crowned dens syndrome

## Abstract

Crowned dens syndrome (CDS) is a rare syndrome of calcium pyrophosphate dihydrate (CPPD) deposition on the odontoid process of the second cervical vertebra leading to unique clinical presentation and radiographical findings. Symptoms usually overlap with more common etiologies, including meningitis, stroke, and giant cell arteritis. Thus, patients struggle with extensive evaluation before diagnosing this uncommon condition. There are few case reports and case series of CDS in the literature. Patients respond well to treatment, but unfortunately, there is a high rate of relapse. Here we present an interesting case of a 78-year-old female patient who presented with acute onset headache and neck pain.

## Introduction

In 1962 advancements in the techniques used to analyze synovial fluid led to the identification of crystals that looked different from urate crystals. These crystals were found in joint fluids aspirated from the affected joints of six arthritic patients and described as rodlike and rhombic with a size of 1 to 25 microns along the longitudinal axis. Unlike urate crystals, they exhibited a weakly positive sign of birefringence by polarized light microscopy. The crystals were identified as a calcium salt of a complex phosphate, closely resembling pyrophosphate. Injection of a suspension of these crystals into the joints of normal humans and dogs produced an acute inflammatory response. A new disease entity was identified, and because of the similarity of the acute attacks to classic acute gouty arthritis and the resemblance of the chronic joint findings to chronic tophaceous gout, the authors named the new condition "pseudogout" [1].

Almost two decades after the identification of pseudogout as a distinct disease entity, crowned dens syndrome (CDS) was described. In this syndrome, calcium pyrophosphate dihydrate (CPPD) deposition on the odontoid process of the second cervical vertebra led to distinct findings on imaging. The calcifications appeared as radiopaque densities of various sizes and shapes, which surrounded the top and sides of the odontoid process like a crown (or a halo) on the head. All of the initially reported five patients experienced severe neck pain and rigidity [2]. Other reported clinical manifestations in the literature are jaw claudication, fever, and mental status changes. These clinical manifestations overlap with more common diseases, including meningitis, stroke, and giant cell arteritis, which makes the diagnosis of crowned dens syndrome a diagnostic challenge for physicians [3]. 

## Case presentation

A 78-year-old woman initially presented with the sudden onset of headache and bilateral neck pain without focal neurologic findings. Her symptoms improved and were attributed to a musculoskeletal etiology. Three weeks later, she required hospital admission for left upper extremity weakness, slurred speech, and posterior headache with radiation of pain to the neck and ears. Other symptoms included right temporomandibular pain, gait instability, night sweats, and decreased appetite. She denied jaw claudication, double vision, or other visual changes. Her past medical history was notable for osteoarthritis requiring knee arthroplasty, hypertension, interstitial cystitis, Alzheimer's disease, and peptic ulcer disease.

The clinical examination revealed a temperature of 98.2 F and a blood pressure of 133/73 mm Hg. She had decreased movement of the cervical spine in all directions but no muscle rigidity, the right temporomandibular joint (TMJ) diminished range of motion (ROM), and tenderness, swelling, and tenderness of the left thumb metacarpal phalangeal joint. She had a slightly diminished sensation to touch in the left upper extremity with no other remarkable findings on her neurologic examination. She did not have tenderness on palpation over the temples bilaterally, and the skin examination was negative for rash.

Diagnostic studies revealed Hb 12.4 g/dl, WBC 9.5 K/uL, platelets 466 K/uL, erythrocyte sedimentation rate (ESR) 108 mm/Hr, C-reactive protein (CRP) 16.8 mg/dl, rheumatoid factor <10 U/mL, cyclic citrullinated peptide (CCP) IgG 10 units (normal), antinuclear antibody (ANA) screen negative, cerebrospinal fluid (CSF) - WBC 4 (0-5), CSF glucose 58, CSF protein 56. Urinalysis (UA) was unremarkable. 

**Figure 1:**
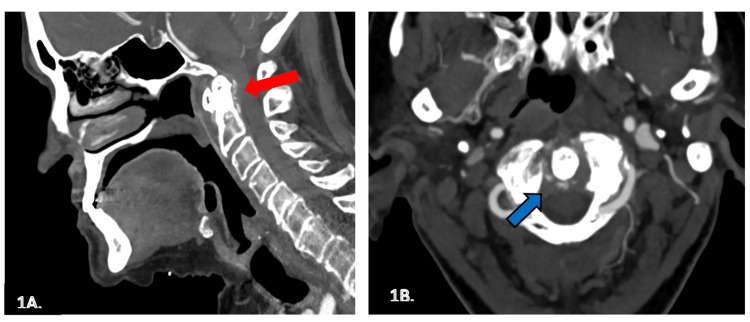
Sagittal (A) and axial (B) views of the cervical spine surrounded by circumferential calcification (arrows) A) Sagittal view of the cervical spine showing calcification posterior to the C2 dens (red arrow). B) Axial view of the cervical spine showing circumferential calcification surrounding the C2 dens-like crown on the head (blue arrow).

MRI cervical spine revealed pannus formation at C1 and diffuse degenerative spondylosis with moderate C5-C6 stenosis. CT angiogram of the neck demonstrated circumferential calcification around the dens consistent with the "crowned dens" manifestation of calcium pyrophosphate dihydrate (CPPD) deposition disease (Figure 1). Plain X-rays demonstrated chondrocalcinosis of the knee and shoulder joints.

While hospitalized, the patient developed a fever of 101.4 F and had transient mental status changes. Initially, diagnostic considerations included infection, cerebrovascular accident, and temporal arteritis. She was started on broad-spectrum antimicrobial therapy while awaiting the results of blood and CSF fluid studies to exclude infection. Cultures were negative for bacterial growth, and antibiotics were stopped. The imaging studies were consistent with the crowned dens syndrome, and prednisone was started. The patient had rapid improvement in her symptoms after starting steroids. She was discharged, and a repeat CRP five months after starting the steroid course was normal at <0.5 mg/dl. She successfully tapered off prednisone and remained stable for six months post-discharge.

The patient was lost to follow up with her primary rheumatologist and did have a relapse one year later. She presented with severe headache, and bilateral neck pain, but no neurological deficits, visual changes, or jaw claudication. She did not have new findings on examination compared to her initial presentation. Repeat ESR was 52mm/hr and CRP was 8.6 mg/dl. At this point, she was evaluated by a different provider, who elected to perform a bilateral temporal artery biopsy. The biopsy result was normal and it was concluded by the other provider that this was a relapse of her CPPD, and she was treated again with a prolonged steroid taper. On her next follow-up appointment two weeks after the second admission, her symptoms improved remarkably, and a repeat CRP was normal at <0.5 mg/dl and ESR was 10 mm/hr.

## Discussion

The etiology of CPPD deposition, in general, is often idiopathic. Occasionally metabolic conditions such as hemochromatosis, Wilson's disease, hyperparathyroidism, and hypomagnesemia may play a precipitating role and should be excluded in patients found to have this crystal arthropathy. When the spine is involved, CPPD crystals are commonly deposited in cervical intervertebral discs, posterior longitudinal ligament, ligamentum flavum, and facet joints. The radiological hallmark-periodontoid deposition is caused by the calcification of the transverse ligament of the atlas. In 90% of cases, deposition is posterior to the odontoid process, with the remainder being either circular, anterior, or lateral [4].

CPPD in any joint is an age-related rheumatological disorder. It is very rare below 50 years of age. The prevalence of chondrocalcinosis in the age range of 60-80 years is 10-15% and is 20% after the age of 80. Despite being a disease of peripheral joints, the spine is commonly involved [5]. A diagnosis of CDS requires the presence of symptoms and should be differentiated from periodontoid calcification, which is a radiological finding without neck pain [6]. CDS occurs in 1.9% of patients with cervical pain and in 21-45% of patients with odontoid calcifications [7]. Somo et al. reviewed CT head that included the odontoid process of 554 patients; 88 had calcification around the odontoid process (15.9%). The prevalence of odontoid calcification was higher in women than men (56 women vs. 32 men), which corresponds to 22% vs. 11% prevalence, respectively, in the given study. Eighteen of the 88 patients developed pseudogout, and 11 among the 18 patients with pseudogout (13% of the total sample) had CDS. Interestingly females, the elderly (80 years and above), and patients with cerebral infarction have a high occurrence of odontoid calcifications [7]. A case-control study by Finckh et al. compared the prevalence of peri-odontoid deposits and cervical pain in 35 diagnosed CPPD patients vs. control. Calcification around the odontoid process was seen in 69% (24 out of 35 CPPD patients) vs. 11% (four patients in the control arm), translating to a six-fold higher occurrence of periodontoid calcifications in CPPD patients. Clinically 34% (12 patients) were asymptomatic, 49% (17 patients) reported mild pain, and 17% (six patients) had severe neck pain; OR for reporting neck pain in CPPD cases vs. clinical control arm was 5.5 [5].

CDS has a very favorable prognosis. Non-steroidal anti-inflammatory drugs (NSAIDs) or corticosteroids are the usual medical therapy for CDS. Goto et al. treated 40 patients with CDS with NSAIDs, prednisolone, or both. The authors observed an early recovery in the NSAID-prednisolone combination group as compared to patients on NSAIDs or prednisolone alone. Nine out of 40 patients had a relapse in nine months and were successfully treated with NSAIDs and steroids. A less effective colchicine-based therapy has been reported. Complications such as cervical cord compression may require surgical intervention. Our patient was treated with steroids and tapered off steroids with no relapse in six months [3,6,8].

## Conclusions

CDS is an uncommon disease entity that is rarely encountered by physicians. It is important to recognize this syndrome because it overlaps with more common syndromes, including meningitis, stroke, and giant cell arteritis. Early recognition and treatment of these syndromes is important, and this can lead physicians to overlook CDS resulting in unnecessary management interventions. Treatment of CDS may include NSAIDs and/or a prolonged steroid taper. It is of utmost importance also to communicate this diagnosis of CDS with the patient's primary care provider, as relapse is observed in up to 25% of patients and can lead to repeating extensive diagnostic evaluation if the treating physician is not aware of the patient's diagnosis.
